# Special Issue: Novel Photoactive Materials

**DOI:** 10.3390/ma11122553

**Published:** 2018-12-15

**Authors:** Maria Vittoria Diamanti

**Affiliations:** Department of Chemistry, Materials and Chemical Engineering “G. Natta” Politecnico di Milano, Via Mancinelli 7, 20131 Milan, Italy; mariavittoria.diamanti@polimi.it; Tel.: +39-02-2399-3137

**Keywords:** photocatalysis, photovoltaics, organic light emitting diodes (OLEDs), TiO_2_, ZnO, density functional theory (DFT)

## Abstract

Photoactivity represents the ability of a material to activate when interacting with light. It can be declined in many ways, and several functionalities arising from this behavior of materials can be exploited, all leading to positive repercussions on our environment. There are several classes of effects of photoactivity, all of which have been deeply investigated in the last few decades, allowing researchers to develop more and more efficient materials and devices. The special issue “Novel Photoactive Materials” has been proposed as a means to present recent developments in the field; for this reason the articles included touch different aspects of photoactivity, from photocatalysis to photovoltaics to light emitting materials, as highlighted in this editorial.

Generally speaking, photoactive materials belong to the huge field of photonics, where materials that actively interact with light are tuned and optimized to achieve effects such as light emission (LEDs and lasers, just to name the most common ones) or light detection, with related signal amplification (e.g., in photomultipliers) and processing operations. Alternatively, they can be used to develop light-sensitive circuits and switches (such as with photoresistors), or more generally, to convert light into an electrical signal (i.e., to build photodiodes). All of these materials and devices are not only subject of research, but already find diffuse applications in our everyday life. 

In particular, the latter case of photodiodes includes devices with huge technological importance in the field of clean energy production, i.e., photovoltaic devices, whose functioning principle is based on the generation of electrical current as a consequence of sunlight absorption, and specifically on the jump of electrons from the valence band to the conduction band when the photoactive material is irradiated with light having energy equal or higher the material bandgap. A similar mechanism, where the photo-generated electrons and holes are exploited in a different way to accelerate chemical reactions, is related to environmental cleanup, as a result of photocatalytic degradation of pollutants in gas and liquid phase. This is currently exploited in air purification devices and water remediation reactors. This same effect can be declined in a similar manner to achieve the so-called self-cleaning effect, where the decomposition of contaminants couples with a further peculiarity of photoinduced superhydrophilicity, which finds relevant applications in building materials due to lower requirements for maintenance and potentially higher durability of self-cleaning materials.

These effects share a common point, that is, the interaction of a material with light, although many different materials are taken into account depending on the effect desired—from elemental semiconductors like silicon, to more complex compounds like CdTe or GaAs, to metal oxides like TiO_2_ and ZnO. Given the broadness of the field, a huge number of works fall within this topic, and new areas of discovery are constantly explored. 

The special issue “Novel Photoactive Materials” has been proposed as a means to present recent developments in the field, and for this reason the eleven articles included touch different aspects of photoactivity: a brief summary of the articles’ content is given in the following. 

The article by Kim et al. [1] reported on the synthesis of 8-hydroxyquinolinato aluminum microwires showing nanoscale photoluminescence properties, with applications as fluorophore in organic light emitting diodes (OLEDs): the enhanced crystal structure and architecture of hexagonal microwires allowed the improvement of optical properties in the material solid state. 

In two articles, the subject of study was photoactive materials for solar cells. In particular, in their feature article, Salado et al. [2] presented a study on the role of interfaces on the performances of multilayer organo-halide perovskite solar cells, with specific reference to the electron selective layer. Indeed, its morphology is a key parameter in determining its performance in extracting charges from the perovskite layer, since energy losses may play a relevant role at this interface. In this respect, a mesoporous or nanostructured interfaces ensured better performance and stability with respect to planar ones, and the recombination mechanism was switched from a mixed mechanism to a trap-limited one. On the other hand, Liu et al. [3] proposed a study on methylammonium lead iodide as light absorber in hybrid solar cells, investigating its geometry, electronic structure, thermodynamic, and mechanical property by density functional theory (DFT) first-principles calculations in order to understand its stability, which is currently a major concern for its application. Caesium (Cs) doping was also taken into account, and the authors demonstrated that its presence can enhance the compound thermodynamic and mechanical stability with no or little influence on its conversion efficiency.

Photocatalysis was the main theme of the other eight articles. A first feature paper by Brzezińska et al. [4] investigated the photocatalytic performance of Cu-ZnO heterojunction co-catalysts, using several zinc oxide formulations obtained with different preparation methods. Photocatalysis tests, focused on the decomposition of 4-chlorophenol in water solution under simulated solar light, allowed the researchers to select the best ZnO synthesis method. Moreover, the possibility to control the oxidation state of the Cu co-catalyst with simple room-temperature treatments was demonstrated. Another feature article by Sanabria Arenas et al. [5] presented a comparison between gas and liquid phase photocatalytic activity of several titanium dioxide (TiO_2_) films grown by anodic oxidation, presenting different morphology. Toluene and rhodamine B (RhB) degradation were selected as model gas and liquid phase reactions, respectively. A strong influence of oxide surface area and crystallinity was observed in both reactions, especially in the gas phase, where the reaction was largely more sensitive to oxide characteristics than the one in liquid phase, showing a less resilient nature of the RhB dye with respect to gas phase toluene. Titanium dioxide was the subject of studies by Cheng et al. [6], Janek et al. [7] and Roveri et al. [8]. The former article proposed the sol-gel production of nitrogen and iron co-doped TiO_2_ catalysts, which were then tested in the degradation of an organic dye, acid orange 7 (AO7). The doping allowed the researchers to obtain high visible light activity, which was further accentuated by choosing a suitably low pH of the AO7 solution, namely pH 3 [6]. In the study by Janek [7], DFT calculations were coupled to physico-chemical analyses to study titanium(IV) oxo-clusters of different formula, allowing the researchers to highlight the influence of substitutes on the measured material band gap and photocatalytic activity, which was estimated by using the degradation of methylene blue (MB) dye as a model reaction. Roveri et al. [8] proposed the use of nanocomposite coatings based on alkylalkoxysilane matrices and TiO_2_ nanoparticles as protective layers on porous stone substrates, and their performances were evaluated to assess the influence of titanium dioxide on the coatings durability, as well as water absorption properties and photocatalytic behavior, studied in the degradation of RhB. The composite coatings allowed the researchers to reach high photocatalytic efficiency while maintaining good protective performances, with only slight effects on the coating durability.

Several other studies involved the evaluation of different material’s photocatalytic activity in the degradation of organic dyes. Tseng et al. [9] chose the degradation of MB to evaluate the photocatalytic activity of their Ag_3_PO_4_ microparticles, which were also tested in the degradation of phenol, both under low-power white-light light-emitting-diode. The high activity obtained was correlated with the microparticles’ large surface area, low recombination rate, and the high charge separation efficiency; further characterization of their photocatalytic activity was carried out in the treatment of environmental water samples polluted with MB, methyl red, acid blue 1, and RhB. Ding et al. [10] used Congo red as model dye to evaluate the photocatalytic efficiency of Bi_2_O_2_(CO_3_)_1−x_S_x_ synthesized by chemical bath precipitation. The control of S content in the precipitation process was observed to play a major role in the material properties, as it broadened the optical absorption of the bismuth oxide-based catalyst towards the visible light region. 

Finally, Palma et al. [11] investigated the possibility to exploit compost-derived bio-based substances to prepare auxiliary hybrid magnetic nanoparticles (HMNPs) for water treatment processes, as a means for an easier recovery of the nanoparticles dispersed in the effluent; their iron-based HMNPs were then used in the photo-Fenton degradation of caffeine in combination with hydrogen peroxide and UV light, whose role was that of generating hydroxyl radicals. The added value of the HMNPs was an increase in the pH of the caffeine solution required to obtain its degradation, and the organic coverage coming from compost also improved the overall degradation reaction. 
